# Three-step matching algorithm to enhance between-group comparability and minimize confounding in comparative effectiveness studies

**DOI:** 10.1038/s41598-021-04014-z

**Published:** 2022-01-07

**Authors:** Chen-Yi Yang, Shihchen Kuo, Edward Chia-Cheng Lai, Huang-Tz Ou

**Affiliations:** 1 Institute of Clinical Pharmacy and Pharmaceutical Sciences, College of Medicine, National Cheng Kung University, 1 University Road, Tainan, 701 Taiwan; 2 Division of Metabolism, Endocrinology and Diabetes, Department of Internal Medicine, University of Michigan Medical School, Ann Arbor, MI USA; 3 Department of Pharmacy, College of Medicine, National Cheng Kung University, Tainan, Taiwan; 4 Department of Pharmacy, National Cheng Kung University Hospital, Tainan, Taiwan

**Keywords:** Epidemiology, Outcomes research

## Abstract

We developed a three-step matching algorithm to enhance the between-group comparability for comparative drug effect studies involving prevalent new-users of the newer study drug versus older comparator drug(s). The three-step matching scheme is to match on: (1) index date of initiating the newer study drug to align the cohort entry time between study groups, (2) medication possession ratio measures that consider prior exposure to all older comparator drugs, and (3) propensity scores estimated from potential confounders. Our approach is illustrated with a comparative cardiovascular safety study of glucagon-like peptide-1 receptor agonist (GLP-1ra) versus sulfonylurea (SU) in type 2 diabetes patients using Taiwan’s National Health Insurance Research Database 2003–2015. 66% of 3195 GLP-1ra users had previously exposed to SU. The between-group comparability was well-achieved after implementing the matching algorithm (i.e., standardized mean difference < 0.2 for all baseline patient characteristics). Compared to SU, the use of GLP-1ra yielded a significantly reduced risk of the primary composite cardiovascular events (hazard ratio [95% confidence interval]: 0.71 [0.54–0.95], *p* = 0.022). Our matching scheme can enhance the between-group comparability in prevalent new-user cohort designs to minimize time-related bias, improve confounder adjustment, and ensure the reliability and validity of study findings.

## Introduction

Inclusion of a broader representative of users exposed to the drug of interest in real-world comparative drug effect studies is crucial to comprehensively assess the drug’s effectiveness and safety, which can ensure the study generalizability. However, when a study involves a comparison between a study (newer) drug and a comparator (older) drug that has been on the market for a long time, there will be many prevalent new-users (PNUs) among the newer study drug group (i.e., those who initiate the newer study drug have been already exposed to the older comparator drug). These PNUs are likely to account for a significant portion of real-life patients using the newer drug, but typically they are all excluded when incident new-user cohort designs (where only “treatment naïve” users of a newer drug can be included) are applied^1^, and therefore, the external validity of study findings to real-world settings is limited.

Although an increasing number of comparative drug effect studies^2–4^ have recognized the importance of including PNUs in analyses, there are two main challenges. First, past exposure to the older drug(s) can be very complicated (i.e., various drug utilization patterns) and difficult for adjustment in analyses. Second, the between-group comparability of baseline patient characteristics and treatment patterns before the initiation of the newer drug could be difficult to achieve. Compared to incident new-users, PNUs are likely to have a wider variety of disease conditions, disease severities, and past drug exposure patterns. Suissa et al.^5^ therefore proposed approaches for baseline characteristics adjustment to achieve the comparability between the newer and older drug groups. They constructed either time- or prescription-based exposure sets to compute the propensity score (PS) of initiating the newer drug and to identify matched individuals from those who received the older drug.

Suissa et al.’s methods only considered past exposure of one specific older drug (i.e., the comparator drug of interest) and adjusted it in the matching process^5^. In real-world settings, however, the newer drug users may have already exposed to multiple older comparator drugs in addition to that specific older comparator drug of interest. In particular, when several older comparator drugs are available on the market before a newer drug is introduced, the patterns of older drug exposure history would be too complex to be measured using the prescription-based exposure set approach proposed by Suissa et al.^5^ Moreover, the prescription-based exposure set, which only measures the past pattern regarding the number of older drugs being used^5^ but does not consider the treatment duration of the older drugs, would raise a concern because the duration of drug exposure is an important indicator for disease progression or severity and can affect study outcomes.

Against this background, we developed a matching scheme with applying the medication possession ratios (MPRs) measure to quantify the complexity of past exposure patterns of all possible older comparator drugs to achieve the baseline characteristics comparability between study groups in PNU cohort designs. In the following, we describe the detailed procedures and illustrate them with a comparative safety study of glucagon-like peptide-1 receptor agonist (GLP-1ra; study drug) versus sulfonylurea (SU; comparator drug) for cardiovascular diseases (CVDs) in a population-based cohort with type 2 diabetes (T2D).

## Methods

The Institutional Review Board of National Cheng Kung University Hospital approved the study before commencement (A-EX-106-013). The informed consents to individual study patients were waived by the Institutional review board of National Cheng Kung University Hospital because the data were de-identified. The study was carried out in accordance with relevant guidelines and regulations.

### Three-step matching algorithm

To achieve the between-group comparability of baseline characteristics, we developed a three-step matching algorithm: (1) matching based on the index date at which the study drug was initiated, (2) matching based on MPRs, which quantified past exposure of all potential older comparator drugs before the index date, and (3) matching based on PS estimated from a variety of confounder patient characteristics measured before or at the index date. Three steps are detailed below.

#### Matching based on the index date

**Figure 1:**
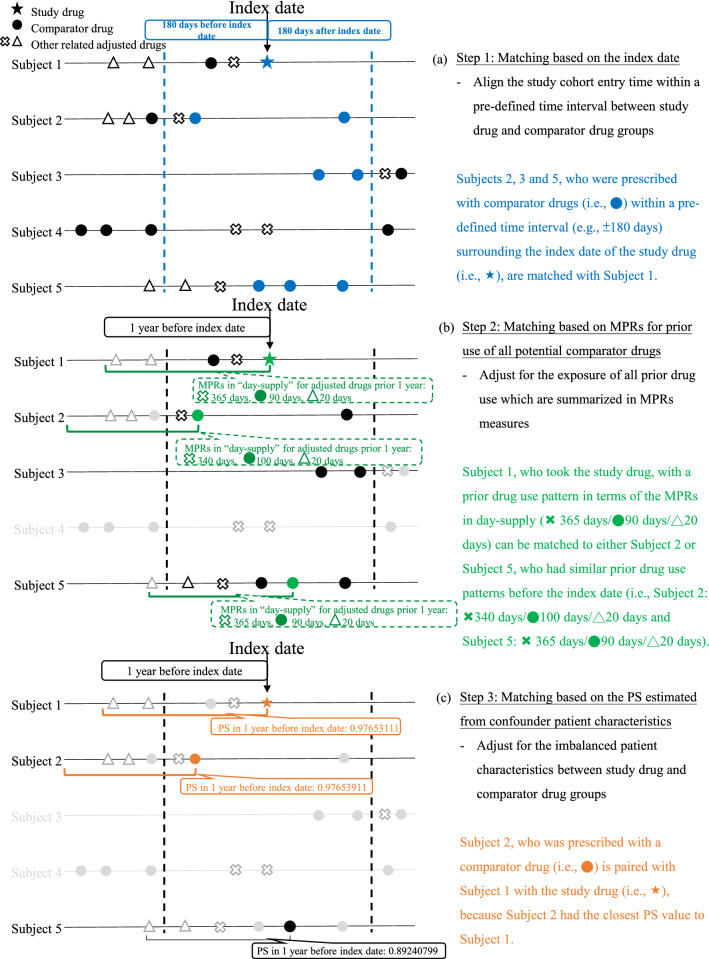
Illustration of proposed matching algorithm: matching based on (**a**) index date with a time interval, (**b**) medication possession ratio (MPR), and (**c**) propensity score (PS).

The index date refers to the initiation date of either the newer study drug or the older comparator drug. We identify the index date of the study drug user along with a pre-defined time interval to match the index date of the comparator drug user to align the study cohort entry time between study groups (Fig. 1a). Considering that comparative drug effect studies often evaluate a newer study drug with an older comparator drug that is not always contemporaneous, balancing index dates would minimize the impact of the unequal study cohort entry time on study outcomes. For example, dipeptidyl peptidase-4 inhibitors (DPP-4i) started being reimbursed in Taiwan’s National Health Insurance (NHI) program in early 2009, but sodium-glucose cotransporter 2 inhibitors (SGLT-2i) were not listed in the NHI formulary until mid-2016. If a head-to-head comparative study is conducted between SGLT-2i and DPP-4i without aligning the index dates, the confounding effect would be probably enlarged given that the circumstances of medication use and healthcare may differ between 2009 and 2016. Due to the granularity of the time scale, we consider a pre-defined time interval for the index date matching between study groups.

#### Matching based on MPRs for prior use of all potential comparator drugs

It is important to balance the patterns of past exposure to study-related medications (i.e., all potential comparator drugs) prior to the index dates between study groups. We propose the utilization of MPRs^6,7^ for measuring the use of all potential comparator drugs prior to the index dates and match these past utilization patterns between study groups (Fig. 1b). Matching on MPRs is to equalize past exposure to all potential comparator drugs, which considers not only the number and type of the drugs but also the duration of the drug exposure. MPR measures can provide an overall profile of past drug treatment history, regardless of drug utilization changes (e.g., discontinuation, or switch to or in combination with another drug). In clinical practice, most patients generally have received diverse treatments with different pharmacological classes and combinations before exposure to the study drugs^8,9^.

#### Matching based on the PS estimated from confounder patient characteristics

Matching based on the PS is used to offset the imbalanced features between study groups^10^. Given a set of covariates identified at the initiation of the study or comparator drugs, the PS summarizes the confounder patient characteristics (e.g., disease conditions and severity, comorbidities, other medication use) and reconstructs the distribution under the overlapping area between study groups to ensure comparability (Fig. 1c)^10^.

## Results

### Illustrative study

An illustrative study for our proposed matching algorithm in a prevalent new-user cohort study for comparative CVD safety of GLP-1ra versus SU in T2D patients is provided in greater detail in Supplementary Method. Briefly, Taiwan’s National Health Insurance Research Database (NHIRD) 2003–2015 was utilized^11^. Study patients included those with T2D diagnosis at age ≥ 18 years during 2003–2014 and with stable GLP-1ra or SU use between 2011 and 2014 after T2D diagnosis. To avoid potential confounding/bias incurred by temporary drug uses, only stable GLP-1ra or SU users were analyzed, as defined in our previous studies^12–17^. Over 99% of 3195 GLP-1ra users had prior glucose-lowering agent (GLA) use, including SU (about 66% of 3195 GLP-1ra users). If using an incident new-user design considering only GLP-1ra users without previous exposure to SU, only 1086 new (naïve) GLP-1ra users can be in the study. This would greatly affect the study generalizability. However, using the PNU cohort design allows us to consider the entire 3195 GLP-1ra users in the study. The study patient selection was illustrated in Supplementary Fig. 1.

There were three matching steps (Fig. 1). First, the index date of a stable user of GLP-1ra was matched with a pre-defined time interval of ± 180 days to the index date of a stable use set of SU. Second, each stable GLP-1ra user was matched with a stable SU use set based on the MPR calculated as the sum of total days of prescriptions/refills for each GLA within one year before the index date. A maximum difference of 90 days (± 45 days) in the MPRs of each class of GLAs between the matched pairs of GLP-1ra stable users and SU stable use set was allowed, which reflects prescription refill pattern under the policy of Taiwan’s NHI program. Third, one-to-one seven-digit greedy matching based on PS (with the maximum distance acceptable for matching of 0.05) was used to balance the confounders between study groups such as patient demographics, diabetes-related complications, comorbidities, and CVD-related medications measured within one year prior to or at the index date (the variables for PS estimation shown in Table 1 Section III). To avoid computation difficulties due to a large variation in the sample sizes, a 10% random sample of the entire SU use sets was used in the matching. Noticeably, all patient characteristics between study groups were balanced after implementing the three-step matching. This supports the enhancement of the between-group comparability by implementing our matching algorithm.

**Table 1:** Baseline patient characteristics before or at the index date and utilization status of glucose-lowering agents among GLP-1ra and SU Groups before and after proposed matching algorithm.

Characteristics^a^	Before matching		After matching	
	GLP-1ra	SU	GLP-1ra	1:1 matched SU
Number of subjects	3195	2,113,699	1573	1573
**Section I (matching step 1)**				
Index date (%)				
Before July 2011	0.47	9.99	0.32	0.70
July 2011–June 2012	10.33	20.93	10.30	10.04
July 2012–June 2013	21.19	21.73	23.33	23.39
July 2013–June 2014	43.57	22.25	41.58	41.51
After June 2014	24.44	25.09	24.48	24.35
**Section II (matching step 2)**				
Number of glucose-lowering agents prescribed one year before index date	2.94	2.34	3.16	3.32
Glucose-lowering agent in one year before index date (MPR, mean ± SD)				
Metformin	0.48 ± 0.43	0.60 ± 0.43	0.62 ± 0.41	0.62 ± 0.41
Sulfonylurea	0.40 ± 0.43	0.82 ± 0.27	0.71 ± 0.35	0.71 ± 0.35
Meglitinide	0.05 ± 0.19	0.01 ± 0.08	0.01 ± 0.09	0.01 ± 0.09
Thiazolidinedione	0.14 ± 0.30	0.09 ± 0.26	0.13 ± 0.30	0.13 ± 0.30
Acarbose	0.15 ± 0.30	0.10 ± 0.27	0.12 ± 0.29	0.12 ± 0.29
DPP-4i	0.41 ± 0.42	0.13 ± 0.30	0.43 ± 0.43	0.43 ± 0.43
Insulin	0.30 ± 0.41	0.03 ± 0.15	0.20 ± 0.36	0.20 ± 0.36
**Section III (matching step 3)**				
Age at index date (year, mean ± SD)	48.61 ± 11.92*	62.37 ± 11.95*	50.2 ± 11.53	51.91 ± 11.98
Males at index date (%)	45.57	53.22	46.92	48.06
Diabetes duration^b^ (year, mean ± SD)	5.99 ± 2.81	5.46 ± 2.77	5.94 ± 2.81	6.48 ± 2.70
Diabetes-related complication (%)				
Retinopathy	17.15	11.26	15.83	17.86
Nephropathy	26.04*	17.55*	25.43	28.29
Neuropathy	13.83	11.61	13.10	14.69
Peripheral vascular diseases	4.54	4.78	3.81	4.70
Cerebrovascular diseases	3.60	7.81	3.56	3.81
Cardiovascular diseases	14.55	18.80	15.00	16.21
Metabolic complications	1.44	0.88	1.02	1.21
Comorbidity (%)				
Hypertension	61.16	66.95	62.75	61.54
Hyperlipidemia	70.14*	57.13*	70.69	71.90
Stroke or TIA	4.48	9.39	4.39	4.70
Heart failure	2.69	3.27	2.29	2.61
Myocardial infarction	1.28	1.33	0.89	1.21
Ischemic heart diseases	11.99	15.28	12.59	13.35
CIC category (%)				
Cancer	4.41	6.88	4.77	5.15
Gastrointestinal	26.23	27.11	25.17	25.43
Musculoskeletal	33.83	40.46	32.04	35.03
Pulmonary	7.86	9.35	7.63	8.58
Substance abuse complexity	2.47	1.65	2.42	3.37
Mental illness	9.45	10.26	8.26	9.98
CVD-related medication (%)				
Lipid modifying agents	67.01*	54.38*	66.50	67.26
α-blockers	3.57	5.20	3.56	3.81
β-blockers	31.92	30.41	31.40	31.85
Agents acting on the renin-angiotensin system	42.25	41.64	44.95	44.37
Diuretics	18.65	17.15	19.71	17.61
CCBs	31.36*	43.31*	33.50	31.28
Antiarrhythmics	1.22	1.85	1.27	1.34
Cardiac glycosides	0.75	1.65	0.95	1.14
Vasodilators used in cardiac diseases	8.26	10.16	7.95	9.09
Anti-platelets	28.17	34.62	27.97	30.71
Anti-coagulants	1.06	1.23	0.95	1.02

GLP-1ra, glucagon-like peptide-1 receptor agonist; SU, sulfonylurea; SD, standard deviation; TIA, transient ischemic attack; CIC, chronic illness with complexity; CVD, cardiovascular disease; CCBs, calcium channel blockers.

*A significant difference in baseline characteristics was found between GLP-1ra and SU groups, indicated by absolute standardized mean difference (SMD) > 0.2; the SMD was less than 0.2 across all variables after matching, indicating balanced baseline characteristics between two drug groups.

^a^All confounders listed above were measured in the year prior to the index date except age and gender, which were measured at the index date; all confounders in section III were included in the estimation of propensity scores. Also, definitions for variables of interest which were used in the propensity score matching are provided in Supplementary Table 2.

^b^Diabetes duration was measured as the time from the first date of type 2 diabetes diagnosis to the index date.

As a result, a lower crude incidence rate of each study outcome was found in GLP-1ra group compared to SU group (Table 2). GLP-1ra use yielded a statistically significant lower risk of the composite CVDs (HR: 0.71, 95% confidence interval [CI] 0.54–0.95) and a statistically non-significant reduced 3-point MACE risk (HR: 0.71, 95% CI 0.44–1.15).

**Table 2:** Incidence rates of cardiovascular events and mortality associated with the stable users of GLP-1ra versus SU^a^.

	GLP-1ra (n = 1573)	1:1 matched SU (n = 1573)
**Composite CVD**^**b**^		
Number of events	75	122
Total person-years in follow-up	2312.08	2727.30
Crude rate (per 1000 person-years)	32.44	44.73
**All-cause mortality**		
Number of events	2	14
Total person-years in follow-up	2372.88	2863.76
Crude rate (per 1000 person-years)	0.84	4.89
**Fatal CVD**		
Number of events	2	8
Total person-years in follow-up	2372.88	2864.25
Crude rate (per 1000 person-years)	0.84	2.79
**MACE**^**c**^		
Number of events	27	45
Total person-years in follow-up	2358.04	2824.41
Crude rate (per 1000 person-years)	11.45	15.93

GLP-1ra, glucagon-like peptide-1 receptor agonist; SU, sulfonylurea; CVD, cardiovascular disease; MACE, major adverse cardiovascular event.

^a^Stable users were those with at least a stable use set of GLP-1ra (or SU), which was defined as at least three sequential refills of GLP-1ra after the index date, with a prescription gap between any two sequential refills of less than 30 days.

^b^Composite CVD was a composite outcome including acute myocardial infarction, ischemic heart disease, heart failure, stroke, cardiogenic shock, sudden cardiac arrest, arteriosclerotic cardiovascular disease, and arrhythmia.

^c^MACE included non-fatal myocardial infarction, non-fatal stroke, and death due to cardiovascular diseases.

## Discussion

With increasing comparative drug effect studies aimed to include PNUs for enhancing the generalizability of study results to real-world settings, the proposed matching algorithm can be a feasible approach to achieve the cohort comparability between study groups for assessing the drug’s effects on study outcomes. Our proposed matching algorithm considers all aspects of potential confounding and bias that may be introduced in analyzing PNUs to enhance the confounder adjustment and ensure the study validity. Specifically, matching based on the index date is essential to align the study cohort entry time between study groups, matching based on MPRs is important to balance past utilization patterns of all possible older comparator drugs between study groups, and matching based on PS is used to adjust for all other potential confounders (e.g., disease severities, comorbidities, other medication use) that could possibly influence study outcomes. Compared to the PNU cohort design by Suissa et al.^5^, where only the two-step matching process is constructed, our proposed three-step matching algorithm provides more comprehensive and precise adjustment to enhance the between-group comparability.

In particular, our algorithm minimizes potential time-related biases (i.e., immortal time bias, time-window bias, time-lag bias), which are common issues in observational studies and could exaggerate the benefits of a study drug of interest^18,19^. Specifically, immortal time and time-window biases can be reduced by aligning the study cohort entry time between study comparison groups (i.e., using the index date matching). Also, the time-lag bias to study estimate is lowered when study comparison groups possess comparable disease stage/severity, which can be achieved by balancing both prior use patterns of all possible older comparator drugs (e.g., GLAs in our illustration study) and confounder patient characteristics between study groups (i.e., applying the MPR and PS matching).

The application of the MPR in our matching algorithm yields the following advantages. First, in real-world practice settings where multiple comparator drugs that could have an impact on study outcomes are available before the study drug is introduced, adjustment based on the prior exposure patterns of all potential comparator drugs is important. In our illustration, there were several GLAs available (e.g., metformin, SU, thiazolidinedione, DPP-4i, and insulin) before GLP-1ra, and some may have potential CVD effects. We found that about 87% of the GLP-1ra users were previously exposed to at least two different GLAs. However, in the study of Suissa et al.^5^, only the past exposure to the specific comparator drug of interest (i.e., SU) was considered in the analysis. Different from it, we further introduce the MPR measure to quantify the past exposure to all comparator drugs and match them between study groups. However, considering the prior exposure to all comparator drugs rather than only a specific one for the matching would downsize the study population. For example, our illustration study identified a final study cohort of 1573 GLP-1ra users from 3195 GLP-1ra users, where most of them were dropped from the MPR matching process. This is because in the real world, the GLP-1ra users usually have had exposure to multiple GLAs before using GLP-1ra, while the SU users generally have not. When we adjusted the past utilization of all different GLAs instead of only the past SU use, some GLP-1ra users who had the complicated GLA use history could not be matched to the SU users with the comparable past GLA exposure and then were unable to be included in the analyses. This trade-off between the study precision by comprehensively adjusting for the past drug use and sample size could be a consideration of applying our matching algorithm.

Second, the pattern (e.g., discontinuation, switch to or combination with other drugs) of the past history of all potential comparator drugs could be very complex, and is thus difficult to quantify and adjust. In this circumstance, the MPR can be a convenient measure to sum the total days of supply within a specific time period (e.g., 365 days) for a given drug, regardless of changes in utilization pattern (i.e., discontinuation, switch to or combination with another drug). Therefore, matching can then be feasibly done according to the MPR values of individual prior drug uses between study drug groups.

Third, the MPR approach considers not only the number and type of drugs but also the length/duration of drug exposure, whereas the original prescription-based exposure set approach only considers the number of drugs being exposed previously^5^. In our illustration, matching based on MPR ensured that the patterns of prior exposure to different GLAs, in terms of their numbers, types, and exposure durations, were balanced between the GLP-1ra and SU groups.

Lastly, the MPR is calculated based on medication refills, which might be a proxy for patients’ health behaviors^20,21^. For example, patients who have high persistence or adherence to medication refills (as indicated by high MPR values) may tend to engage in healthy behaviors (e.g., regular exercise, healthy diet) compared to those that do not (as indicated by low MPR values). With this regard, the comparative effectiveness of study drugs (e.g., GLP-1ra versus SU) on study outcomes (e.g., CVDs) might be confounded by this healthy user effect. Hence, the adjustment based on MPR measures might allow researchers to control for potential healthy user bias/effect.

The proposed matching algorithm was illustrated in a study of the comparative safety of GLP-1ra versus SU for CVDs and mortality, which has been previously investigated. Recent meta-analyses of existing trials indicate either statistically significant or non-significant lower risks for all-cause mortality and fatal CVDs associated with GLP-1ra versus SU use^22,23^. Three cohort studies based on the incident new-user cohort design also showed a statistically non-significant lower risk of the composite CVDs with GLP-1ra compared to SU^24–26^. The findings of favorable cardiovascular outcomes of GLP-1ra versus SU observed in our illustration study are generally consistent with those in previous studies including several cardiovascular outcome trials of GLP-1ra versus placebo^27–30^, which supports the validity of our approach and may suggest that cardiovascular benefits reported in clinical trials could be extended to clinical practice settings. However, unlike previous studies considering either highly selective trial populations or only incident new users of GLP-1ra, including a broader spectrum of GLP-1ra users and applying more rigorous matching methodology in this study may guarantee us to reveal valid and generalizable results. Nonetheless, more real-world studies are warranted to corroborate the comparative effectiveness and safety of GLP-1ra versus other GLAs to inform T2D management. Our proposed approach would therefore be handy for this task.

There are several limitations inherent to the developed approach. First, in real-world practice, several factors such as physicians’ prescribing tendency, drug adverse effects, or financial considerations may lead to patients’ early discontinuation or non-adherence of medications. However, the validity of effectiveness estimates in the real-world drug studies could be affected by the bias or confounding introduced from these accidental use or non-adherence issues. Therefore, our illustration only considered the stable study drug users^12–17^ in the analyses to ensure accurate assessment and implications when relating medication treatment to health outcomes. Second, our matching algorithm might be computationally demanding because it is operated on the basis of matching drug use sets of the comparator drug (i.e., SU in our illustration). However, this issue could be overcome by random sampling as shown in our illustration. Also, if the sample size of the comparator drug group is much larger than that of the study drug group, computing the PS based on the logistic regression analysis or Cox model would be problematic due to such a large disparity in sample size between the two drug groups^31,32^. As shown in our illustration, we recommend performing a random sampling procedure (i.e., 10%, 30%, 50%, and 100%) on the comparator drug group to inspect the fitness of the distribution of PS (i.e., kernel density curves) between the two groups. Third, the influence of reusing comparator drug use sets remains to have future research, in particular the potential impact of non-independence in the estimation of CIs, which could further require techniques based on bootstrap sampling^5^. Fourth, a number of medication use measures exists in the literature. For efficient computation, MPR was chosen in this study. The impact of adopting different medication utilization measures (e.g., proportion of days covered) on study results deserves future research. Lastly, some pre-defined time periods were applied in our illustration (e.g., index date ± 180 days as the time interval to provide a one-year time frame for matching, and a one-year period before the index date for estimating PS), which could be further examined for their appropriateness.

## Conclusions

For comparative drug effectiveness studies involving PNUs, we developed a feasible and precise three-step matching algorithm that can be used to enhance the between-group comparability, minimize time-related bias issues, and improve confounder adjustment. The illustrative study with the application of the three-step matching algorithm revealed that compared to a SU, GLP-1ra use was associated with a significant lower cardiovascular disease risk among a real-world T2D population.

## Supplementary Information

Supplementary Information.
