# Supplementary material for: Three-step matching algorithm to enhance between-group comparability and minimize confounding in comparative effectiveness studies
Source: Sci Rep. 2022 Jan 7;12:214. doi: 10.1038/s41598-021-04014-z (PMC8741962; doi:10.1038/s41598-021-04014-z)
Supplement: Supplementary file 1 — Supplementary Information. [file 41598_2021_4014_MOESM1_ESM.docx]

**Supplementary Method**

Cohort identification

Taiwan’s National Health Insurance Research Database (NHIRD) 2003-2015 was utilized [11]. The cohort entry time of 2011-2014 was used because glucagon-like peptide-1 receptor agonist (GLP-1ra) was not reimbursed by the National Health Insurance (NHI) program until 2011 and this design allowed at least one-year follow-up for patients identified on 2014/12/31. Patients who met the following criteria were included: (1) newly diagnosed with type 2 diabetes (T2D) during 2003-2014, confirmed by one inpatient record with T2D (International Classification of Disease, Ninth. Revision, Clinical Modification: 250.x0 or 250.x2, where x=0-9), two outpatient records with T2D, or one outpatient record with T2D with at least one prescription refill record of a glucose-lowering agent (GLA) within a given year and without any T2D diagnoses in the preceding year, (2) age ≥ 18 years, and (3) stable use of GLP-1ra or SU during 2011-2014 after T2D diagnosis.

To avoid potential confounding/bias incurred by temporary drug uses (e.g., short-term or accidental use), we only considered stable users of GLP-1ra (or SU) in analyses. Stable users were those with at least a stable use set of GLP-1ra (or SU), which was defined as at least three sequential refills of GLP-1ra after the index date, with a prescription gap between any two sequential refills of less than 30 days. The procedures and rationale of defining stable users were detailed in our previous studies [12-17]. A stable user of GLP-1ra (or SU) could have multiple stable use sets of GLP-1ra over time. There were 3,195 stable users of GLP-1ra and 773,026 stable users of SU identified. It’s noted that over 99% of the GLP-1ra users had previously been prescribed with glucose-lowering agents (GLAs), including SU (about 66% of the 3,195 GLP-1ra users had previously used SU). If using an incident new-user design that considers only GLP-1ra users without previous exposure to SU, we can include only 1,086 new (naïve) GLP-1ra users for consideration in the study. This would greatly affect the study generalizability. However, using the PNU cohort design allows us to consider the entire 3,195 GLP-1ra users in the study. A flow diagram for selection of study patients is provided in Supplementary Figure 1.

Matching algorithm

As shown in Figure 1(a), in the first step, the index date of a stable user of GLP-1ra was matched with a pre-defined time interval of ±180 days to the index date of a stable use set of SU. To simply our analyses, each GLP-1ra user only had one index date, which was defined as the first date of the first stable use set. We allowed each SU stable use set to be reused as matched samples to GLP-1ra users to increase the possibility of identifying the best matched use set of SU for GLP-1ra users. A total of 21,135,785 stable use sets of SU from 773,026 stable users of SU were identified for 2011-2014. In the second step (Figure 1(b)), each stable user of GLP-1ra was matched with a stable use set of SU based on the MPR calculated as the sum of total days of prescriptions/refills for each GLA within one year before the index date, including metformin, SU, meglitinide, thiazolidinedione, acarbose, DPP-4i, and insulin. Notably, we allowed a maximum difference of 90 days (±45 days) in the MPRs of each class of GLAs between the matched pairs of GLP-1ra stable users and SU stable users. The rationale for applying this 90-day time window in the MPR is that the longest duration for chronic prescription drugs (e.g., GLAs) is three months according to the policy of Taiwan’s NHI program, so patients may have a maximum time gap between two consecutive prescription refills of 90 days approximately. Lastly, one-to-one seven-digit greedy matching based on PS (with the maximum distance acceptable for matching of 0.05) was used to balance the confounders between study groups such as patient demographics, diabetes-related complications, comorbidities, and CVD-related medications measured within one year prior to or at the index date (Figure 1(c)). The variables for estimating the PS were detailed in Table 1 section III.

To avoid computation difficulties due to a large variation in the sample size between study groups, a 10% random sample of the entire SU stable use sets was used in the matching algorithm. In our analyses, we used 3,195 stable users of GLP-1ra and 2,113,699 stable use sets from 595,561 stable users of SU. Table 1 shows the patients’ characteristics before and after the matching algorithm. Noticeably, it shows that several baseline characteristics were statistically different between study groups with a standardized mean difference > 0.2 before the matching, while all of these characteristics were balanced between study groups after the matching. This supports the enhancement of the between-group comparability by implementing this matching algorithm.

Statistical analyses

Primary outcomes were composite CVD events and three-point major adverse cardiovascular events (MACE) (disease definitions are provided in Supplementary Table 1). Secondary outcomes included all-cause mortality and fatal CVDs. The follow-up period for each study subject was from the index date until the occurrence of study outcomes, the discontinuation of GLP-1ra or SU, death, dropout/lost follow-up from the NHI program, or the end of 2015, whichever came first. The incidence rate of study outcomes was calculated as the total number of events over the follow-up period divided by the number of person-years at risk. Cox models were used to compare the risks of study outcomes between the two matched study groups.

Study results

Table 2 shows the incidence rates of CVDs and mortality associated with the use of GLP-1ra versus SU. The crude incidence rate of each study outcome was lower in the GLP-1ra group. The results of Cox model analyses demonstrate that the use of GLP-1ra yielded a statistically significant lower risk of the composite CVDs (hazard ratio: 0.71, 95% confidence interval: 0.54-0.95) and a statistically non-significant reduced risk of 3-point MACE (0.71, 0.44-1.15).

**Supplementary** **Figure 1.** Flow diagram for selection of study patients

Population insured in Taiwan’s National Health Insurance program from 2003-2015 (n=22,900,726)

Newly-diagnosed type 2 diabetes mellitus from 2003-2014

(n=2,338,292)

Patients who were ever prescribed with GLP-1ra during 2010-2014

(n=4,125)

Patients who were ever prescribed with SU during 2010-2014

(n=929,240)

Patients who were stably prescribed with GLP-1ra during 2010-2014

(stable users n=3,195)

(stable user sets N=3,195)

Patients who were stably prescribed with SU during 2010-2014

(stable users n=773,026)

(stable user sets N=21,135,785)

Excluded:

- Gender undefined (n=10)
- Diabetes diagnosed before using GLP-1ra (n=16)
- Patients who are not stably used GLP-1ra (n=904)

Excluded:

- Gender undefined (n=239)
- Diabetes diagnosed before using SU (n=2,353)
- Patients who are not stably used SU (n=153,622)

Patients who were stably prescribed with SU during 2010-2014 with 10% sampling

(stable users n=595,561)

(stable user sets N=2,113,699)

Abbreviations: GLP-1ra, glucagon-like peptide-1 receptor agonist; SU, sulfonylurea.

**Supplementary Table 1.** International Classification of Disease, 9^th^ edition, Clinical Modification (ICD-9-CM) codes used to define study outcomes of interest

| **Outcome** | **ICD-9-CM disease code** | **ICD-9-CM procedure code** | **Data source** |
| --- | --- | --- | --- |
| Cardiovascular diseases^a,b,c^ | | |  |
| Acute myocardial infarction | 410 | --- | ER or inpatient |
| Stroke | 430-437, V12.54 | 00.61, 00.63, 38.11, 38.12 | ER or inpatient |
| Heart failure | 428 |  | ER or inpatient |
| Ischemic heart diseases | 411, 413, 414, V45.81, V45.82 | 00.66^c^, 36.0, 36.1, 36.2, 36.3, 36.9, 88.5 | ER or inpatient |
| Cardiogenic shock | 785.51 | --- | ER or inpatient |
| Sudden cardiac arrest | V12.53 | --- | ER or inpatient |
| Arteriosclerotic cardiovascular diseases | 429.2 | --- | ER or inpatient |
| Arrhythmia | 426, 427 | --- | ER or inpatient |
| Major adverse cardiovascular event |  |  |  |
| Death | --- | --- | Deceased records |
| Non-fatal myocardial infarction | 410 | --- | ER or inpatient |
| Non-fatal stroke | 430-437, V12.54 | 00.61, 00.63, 38.11, 38.12 | ER or inpatient |

Abbreviation: ER, emergency room.

a. *Am J Manag Care* 2012;18:721-726.

b. *Nutrition, Metabolism and Cardiovascular Diseases* 2014;24:10-17.

c. *Cardiovascular Diabetology* 2014;13:3.

**Supplementary Table 2.** International Classification of Disease, 9^th^ edition, Clinical Modification (ICD-9-CM) codes used to define variables of interest which were used in propensity score matching

| **Variable*** | **ICD-9-CM disease code** | **ICD-9-CM procedure code** |
| --- | --- | --- |
| Complications and comorbidity^a,b,c,d^ |  |  |
| Retinopathy | 361, 362, 369, 250.5, 379.23 | --- |
| Nephropathy | 580, 581, 582, 583, 585, 586, 250.4, 593.9 | --- |
| Neuropathy | 354, 355, 458, 250.6, 356.9, 358.1, 951.0, 951.1, 951.3, 713.5, 357.2, 337.0, 337.1, 564.5, 536.3, 596.54 | --- |
| Peripheral vascular disease | 250.7, 040.0, 785.4, 442.3, 892.1, 707.1, 444,22 | --- |
| Cerebrovascular diseases | 431, 433, 434, 435, 436, 437, | --- |
| Cardiovascular diseases | 410, 411, 412, 413, 414, 428, 440, 441, 4271, 4273, 4274, 4275, 4292 | --- |
| Metabolic complications | 250.1, 250.2, 250.3 | --- |
| Hypertension | 401, 402, 403, 404, 405 | --- |
| Hyperlipidemia | 272 | --- |
| Stroke | 430-434, 436-438, V12.54 | 00.61, 00.63, 38.11, 38.12 |
| Heart failure | 428 | --- |
| Myocardial infarction | 410, 412 | --- |
| Ischemic heart diseases | 411, 413, 414, V45.81, V45.82 | 360, 361, 362, 363, 369,  885, 00.66 |
| Chronic illness with complexity^e,f^ |  |  |
| Cancer | 140-165, 170-176, 179, 180-208, 230-239 |  |
| Gastrointestinal illness | 531-534, 555, 556, 562.11, 562.13, 070, 571, 572.2, 572.3, 572.4, 572.8, 574-576 |  |
| Musculoskeletal illness | 715, 714, 274, 712, 716, 719.05, 719.15, 719.25, 719.35, 719.45, 719.55, 719.65, 719.75, 719.85, 719.95, 726.5, 733.14, 733.15, 733.42, 820, 720, 721.3, 721.42, 722.10, 722.52, 722.73, 722.83, 722.93, 724.02, 724.2-724.9 |  |
| Pulmonary illness | 493, 491, 492, 496 |  |
| Substance abuse | 304.00, 304.01, 304.02, 304.03, 304.70, 304.71, 304.72, 265.2, 291.0, 291.1, 291.2, 291.3, 291.4, 291.5, 291.9, 303, 305.0, 357.5, 425.5, 535.3, 571.2, 571.3, 790.3, 292, 304, 305.2-305.9, 305.1 |  |
| Mental illness | 296.2, 296.3, 311, 300.4, 296.0, 296.1, 296.4-296.9, 309.81, 295, 300.0, 300.2, 300.3 |  |

*Sources of data are from inpatient, outpatient and emergency room records.

1. *Am J Manag Care* 2012;18:721-726.
2. *Nutrition, Metabolism and Cardiovascular Diseases* 2014;24:10-17.
3. *Cardiovascular Diabetology* 2014;13:3.
4. *Diabetes Care*. 2018 May;41(5):917-928.
5. *J Gen Intern Med*. 2007;22 Suppl 3:408-418.
6. *Medical care*. 2015;53(2):106-115.
